# Determination of the available energy values and amino acid digestibility of *Flammulina velutipes* stem waste and its effects on carcass trait and meat quality fed to growing-finishing pigs

**DOI:** 10.1186/s40104-020-00449-y

**Published:** 2020-05-04

**Authors:** Xuzhou Liu, Bo Zhang, Hansuo Liu, Gang Zhang, Jinbiao Zhao, Ling Liu, Xiangshu Piao, Hui Song, Shuai Zhang, Yu Li

**Affiliations:** 1 Institute of Mycology, Engineering Research Center of Chinese Ministry of Education for Edible and Medicinal Fungi, Jilin Agricultural University, Changchun, 130118 China; 2 State Key Laboratory of Animal Nutrition, Ministry of Agriculture Feed Industry Centre, China Agricultural University, Beijing, 100193 China

**Keywords:** Amino acid digestibility, Available energy, Fiber, *Flammulina velutipes* stem waste, Growing-finishing pigs, Growth performance, Meat quality

## Abstract

**Background:**

*Flammulina velutipes* stem waste (FVS) is the by-product of mushroom industry. The objectives of this study were to determine the available energy and amino acid digestibility of FVS fed to pigs, and to evaluate the effects of dietary FVS inclusion on growth performance, biochemical profile of serum, fecal short chain fatty acid (SCFA) concentration, carcass traits, meat quality, intestinal morphology and microflora of pigs. In Exp. 1, twelve crossbred barrows with initial body weight (IBW) of 37.48 ± 4.31 kg were randomly allotted to 2 dietary treatments, including a corn basal diet and an experimental diet containing 24.35% FVS. In Exp. 2, twelve barrows fitted with an ileal T-cannula (IBW: 32.56 ± 1.67 kg) were randomly allotted to 2 dietary treatments, which included a N-free diet and an experimental diet containing 40.0% FVS. In Exp. 3, ninety growing pigs (IBW: 63.98 ± 6.89 kg) were allotted to 1 of 3 treatment diets for 63 d, including a basal diet and 2 experimental diets with 2.5% and 5% FVS, respectively.

**Results:**

The digestible energy (DE) and metabolizable energy (ME) of FVS were 4.58 and 4.06 MJ/kg on dry matter basis, respectively, and the standardized ileal digestibility (SID) of indispensable AAs ranged from 17.50% to 59.47%. Pigs fed diets with 2.5% FVS showed no difference on average daily gain (ADG) and gain to feed ratio (G/F). Although dietary 5% FVS inclusion impaired apparent total tract digestibility (ATTD) of organic matter and gross energy, it elevated the SCFA concentration (*P* ≤ 0.04) in gut and antioxidant capacity in serum. In addition, dietary FVS inclusion depressed the backfat thickness (*P* = 0.03) in pigs. The *longissimus dorsi* muscle of pigs fed FVS revealed higher n-3 polyunsaturated fatty acid concentration and optimized fatty acid composition. Dietary 2.5% FVS inclusion also improved the intestinal development and health by increasing the villius height to crypt depth ratio (V/C) in jejunum (*P* < 0.01), and promoting microbial diversity and beneficial microbiota proliferation.

**Conclusions:**

It is feasible to include moderate content of FVS as an unconventional fiber ingredient in diet of growing-finishing pigs.

## Background

*Flammulina velutipes* (FV) is a kind of edible mushroom that is popular especially in Asia. In 2015, the total output of FV in China exceeded 758.3 thousand tons [1]. The by-product of FV *– Flammulina velutipes* stem waste (FVS), has also been produced in large amounts, and the common direct disposal of FVS turned out to be a great threat to our environment and also a huge waste of resources. According to the previous investagation, the average processing cost (mainly due to the electricity consumption) of the FVS powder in China was 43 dollars per ton, while the cost of the raw materials (crude FVS) is almost negligible at present [2]. Thus, the moderate inclusion of FVS in animal diets could greatly reduce the feed cost.

In recent years, some studies have explored the utilization of FVS in broiler chicken diets. For example, Wang et al. [3] and Mahfuz et al. [4] reported that dietary FVS inclusion improved the growth performance of broilers, and also increased the interleukin-2 (IL-2), interleukin-4 (IL-4) and S-immunoglobulin A (S-IgA) concentrations in serum, the short chain fatty acid (SCFA) concentration in gut, and the villus height to crypt depth ratio (V/C) in the intestine of broilers. However, there is no study focusing on the utilization of FVS as a feed ingredient in diets of growing-finishing pigs.

Therefore, the objectives of this study were to determine the digestible energy (DE), metabolizable energy (ME) values, and the apparent ileal digestibility (AID) and standardized ileal digestibility (SID) of amino acids (AAs) of FVS fed to growing pigs, to determine the effects of dietary FVS inclusion at different inclusion levels (2.5% or 5%) on growth performance, apparent total tract digestibility (ATTD) of nutrients, biochemical profiles in serum and fecal short chain fatty acid (SCFA) concentration in growing-finishing pigs, and to determine the effects of dietary FVS inclusion on carcass characteristics, meat quality, intestinal morphological structure and microflora in finishing pigs.

## Materials and methods

All experiments were carried out in accordance with the Chinese Guidelines for Animal Welfare and Experimental Protocol, and received prior approval by the Animal Care and Use Committee of China Agricultural University (ID: SKLABB-2010-003).

The animal trials of Exp. 1 and 3 were carried out in the FengNing Swine Research Unit of China Agricultural University (Academician Workstation in Chengdejiuyun Agricultural & Livestock Co. Ltd). The animal trial of Exp. 2 was conducted in the Metabolism Laboratory of the Ministry of Agriculture Feed Industry Centre in China Agricultural University (Beijing, China). The crude FVS was provided by *Ficus altissima* Biotechnology Company (Changchun, China), and was processed in Jilin Green Biological Technology Company (Siping, China). Specifically, FVS was dried using a triple-pass rotary drum dryer machine (65 °C) and then ground in a hammer mill using a 2-mm screen. The same batch of FVS was used for all experiments. The chemical compositions of FVS was shown in Table 1.

**Table 1:** Analyzed nutrient content of *Flammulina velutipes* stem waste (%, as-fed basis)^a^

Item	FVS
Dry matter	89.70
Crude protein	13.69
Gross energy, MJ/kg	15.88
Ash	8.60
Ether extract	1.60
Crude fiber	15.30
Neutral detergent fiber	34.06
Acid detergent fiber	18.41
Total phosphorus	0.45
Calcium	0.58
Essential amino acids	
Lysine	0.57
Threonine	0.47
Methionine	0.14
Tryptophan	0.15
Leucine	0.61
Valine	0.46
Phenylalanine	0.47
Isoleucine	0.44
Arginine	0.42
Histidine	0.23
Nonessential amino acids	
Glutamic acid	1.61
Tyrosine	0.39
Serine	0.43
Glycine	0.45
Proline	0.49
Cysteine	0.13
Alanine	0.71
Aspartic acid	0.84
Total amino acids	9.01

^a^All values are the results of an analysis conducted in duplicate. FVS: *Flammulina velutipes* stem waste

### Exp. 1: DE and ME contents of FVS

Twelve crossbred barrows (Duroc × Landrace × Yorkshire) with initial body weight (IBW) of 37.48 ± 4.31 kg were assigned to 2 treatment diets in a completely randomized design with 6 replicated pigs per treatment. The treatment diets included a basal diet formulated to contain 97.4% corn and 2.6% of vitamins and minerals, and an experimental diet formulated by replacing 25% of corn in the basal diet by FVS (Table 2).

**Table 2:** Ingredients and analyzed AA compositions of the experimental diets used in Exp. 1 and 2 (%, as-fed basis)

Item	Exp. 1		Exp. 2	
	Basal diet	FVS diet	FVS diet	N-free diet
Ingredients				
Corn	97.40	73.05	–	–
Corn starch	–	–	40.80	76.80
Sucrose	–	–	12.00	12.00
Cellulose acetae	–	–	–	4.00
FVS^a^	–	24.35	40.00	–
Soybean oil	–	–	3.00	3.00
Dicalcium phosphate	0.90	0.90	2.00	2.00
Limestone	0.90	0.90	0.80	0.80
Sodium chloride	0.30	0.30	0.50	0.50
Choline chloride	–	–	0.10	0.10
Chromic oxide	–	–	0.30	0.30
Vitamin and mineral premix^b^	0.50	0.50	0.50	0.50
Analyzed nutrient levels				
Dry matter	86.29	86.81	89.66	89.23
Crude protein	7.52	7.95	6.01	0.76
Organic matter	97.07	94.98	93.17	94.67
Essential amino acids, g/kg				
Lysine	–	–	2.20	0.08
Methionine	–	–	0.50	–
Threonine	–	–	2.00	0.19
Tryptophan	–	–	0.60	–
Valine	–	–	1.70	0.27
Leucine	–	–	5.20	0.50
Isoleucine	–	–	1.40	0.06
Phenylalanine	–	–	7.70	0.22
Histidine	–	–	0.80	0.17
Arginine	–	–	1.90	0.07
Nonessential amino acids, g/kg				
Tyrosine	–	–	7.20	0.07
Serine	–	–	1.90	0.20
Glutamic acid	–	–	7.10	0.69
Proline	–	–	1.90	0.29
Glycine	–	–	1.70	0.23
Alanine	–	–	2.40	0.28
Cysteine	–	–	0.50	–
Aspartic acid	–	–	3.70	0.26

^a^FVS: *Flammulina velutipes* stem waste

^b^Vitamin and mineral premix provided the following quantities of vitamins and minerals per kg of diet: vitamin A, 5512 IU; vitamin D_3_, 2200 IU; vitamin E, 30 IU; vitamin K_3_, 2.2 mg; vitamin B_12_, 27.6 μg; riboflavin, 4 mg; *D*-pantothenic acid, 14 mg; folic acid, 0.7 mg; thiamine, 1.5 mg; pyridoxine, 3 mg; biotin, 44 μg; Mn 40 mg (as manganese oxide); Fe, 75 mg (as iron sulfate); Zn, 75 mg (as zinc oxide); Cu, 100 mg (as copper sulfate); I, 0.35 mg (as potassium iodide) and Se, 0.3 mg (as sodium selenite)

All pigs were individually placed in stainless steel metabolism crates (1.4 m × 0.45 m × 0.6 m) equipped with a feeder, a nipple drinker and fecal collection trays, and were housed in an environmentally controlled room with the temperature maintained at 23 ± 2 °C. Pigs were provided ad libitum access to water and the daily feed equivalent to 4% of their initial body weight measured at the beginning of the animal trial, and diet was divided into two equal meals supplied at 08:30 and 16:30 h each day [5].

Before the animal trial, pigs were allowed a 5-d period to adapt to metabolic crates and fed a commercial diet. The animal trial lasted for 12 d, including 7 d for adaption to the experimental diets and 5 d for the total collection of feces and urine using a time-based collection procedure. During the collection days, feed refusals and spillage were collected twice daily and immediately dried (65 °C for 8 h) and weighed. Feces were collected from each metabolism crate when appeared in the collection tray and were subsequently stored at − 20 °C. Urine was collected separately in a bucket containing 50 mL of 6 mol/L HCl, and all the buckets were placed under the metabolism crates [6]. A volume of 10% of the total-collected urine per day was stored at − 20 °C. At the end of the experiment, feces and urine samples were subsequently thawed, pooled by pig, homogenized, and subsampled. Then fecal subsamples were dried at 65 °C in a drying oven for 72 h. Four mL urine sample was dropped into crucibles with quantitative filter paper and then dried at 65 °C in a drying oven for 8 h for further analysis of gross energy [7].

### Exp. 2: AA digestibility of FVS

Twelve crossbred barrows (Duroc × Landrace × Yorkshire) with IBW of 32.56 ± 1.67 kg were fitted with T-cannulas at the terminal ileum according to the method of Stein et al. [8]. Pigs were individually housed in stainless steel metabolism crates (1.4 m × 0.45 m × 0.6 m) located in a temperature-controlled room (23 ± 2 °C). All barrows were fed 1 of 2 diets in a completely randomized design with 6 replicated pigs per diet. The treatment diets included a N-free diet containing 76.8% corn starch and 12% sucrose, which was used to evaluate the basal ileal endogenous N and AAs losses [9], and an experimental diet based on corn starch (40.8%) and sucrose (12%) supplemented with 40% FVS as the only source of protein and AA (Table 2). In addition, 0.3% chromic oxide was included in all diets as an indigestible marker. Vitamins and minerals were supplemented in all diets to meet or exceed the recommended nutrient requirements for growing pigs in NRC [10]. All pigs were provided ad libitum access to water and the daily feed equivalent to 4% of their initial body weight, which was divided into two equal meals supplied at 08:00 and 16:00 h each day.

After a 15-d recovery postoperative period, all pigs were allowed a 7-d period to adapt to the environment and fed a commercial diet. Then pigs were fed the treatment diets for a 10-d period, including a 8-d dietary acclimation period and a 2-d digesta collection period, which lasted for 9 h daily beginning at 08:00 h [8]. On d 9 and 10, plastic bag was attached to the barrel of the cannula. The bags were removed whenever they were full of digesta and then stored at − 20 °C to prevent bacterial degradation in the digesta. At the end of the collection period, all the samples were thawed, mixed by pig, subsampled, and lyophilized in a vacuum freeze dryer (Tofflon Freezing Drying Systems, Shanghai, China).

### Exp. 3: growth trial of FVS and sample collection

Ninety growing pigs (Duroc × Landrace × Yorkshire) with IBW of 63.98 ± 6.89 kg were selected from a commercial herd, and then assigned to 3 treatments in a completely randomized design. Each treatment diet was fed to 6 replicated pens, with 5 pigs (3 barrows and 2 gilts or 2 barrows and 3 gilts) per pen. The treatment diets included a control diet and 2 experimental diets formulated by supplementing with 2.5% or 5% FVS, respectively (Table 3). All the diets were formulated based on the DE value estimated from Exp. 1, and the SID AAs concentration derived from Exp. 2. Experimental diets were carried out to meet the nutrient requirements for pigs in different phases recommended by NRC [10]. The DE, SID Lys, SID Met, SID Thr and SID Trp in all 3 diets were kept the same. All diets were supplied with 0.3% chromic oxide as an indigestible marker in the last 2 weeks of each phase.

**Table 3:** Ingredients and calculated nutrient levels of the experimental diets used in Exp. 3 (%, as-fed basis)^a^

Item	Growing phase: 50 to 75 kg			Growing-finishing phase: 75 to 100 kg			Finishing phase: 100 to 135 kg		
	Control	2.5% FVS	5% FVS	Control	2.5% FVS	5% FVS	Control	2.5% FVS	5% FVS
Ingredients									
Corn	77.89	74.24	70.65	78.74	75.08	71.35	79.49	75.86	72.24
Soybean meal	18.00	18.00	18.00	18.00	18.00	18.00	18.00	18.00	18.00
FVS	–	2.50	5.00	–	2.50	5.00	–	2.50	5.00
Soybean oil	0.58	1.74	2.86	0.24	1.40	2.60	–	1.14	2.30
Dicalcium phosphate	1.18	1.18	1.18	0.95	0.95	0.95	0.66	0.66	0.66
Limestone	0.73	0.69	0.66	0.70	0.70	0.70	0.67	0.65	0.60
Sodium chloride	0.35	0.35	0.35	0.35	0.35	0.35	0.35	0.35	0.35
*L*-Lysine·HCl	0.34	0.35	0.35	0.19	0.19	0.20	0.03	0.04	0.04
*DL*-Methionine	0.02	0.03	0.03	–	–	0.01	–	–	0.01
*L*-Threonine	0.09	0.10	0.10	0.03	0.03	0.04	–	–	–
*L*-Tryptophan	0.02	0.02	0.02	–	–	–	–	–	–
Chromic oxide	0.30	0.30	0.30	0.30	0.30	0.30	0.30	0.30	0.30
Vitamin mineral premix^b^	0.05	0.05	0.05	0.05	0.05	0.05	0.05	0.05	0.05
Analyzed nutrient levels									
Dry matter	87.04	86.89	87.41	86.68	86.88	87.16	86.68	86.80	86.87
Crude protein	13.70	14.08	13.89	13.40	13.49	11.18	11.92	13.28	13.85
Gross energy, MJ/kg	16.03	16.29	16.56	15.90	16.20	16.45	15.87	16.13	16.37
Ash	4.20	4.36	4.49	3.84	4.14	4.48	3.75	3.78	4.43
Ether extract	2.08	3.63	4.29	2.55	4.27	4.00	3.19	3.70	3.77
Neutral detergent fiber	10.99	11.17	13.55	11.47	10.26	11.86	10.42	12.33	13.17
Acid detergent fiber	3.64	4.27	5.41	3.85	4.05	5.08	3.65	4.40	4.69
Calculated nutrient levels^c^									
ME, MJ/kg	14.05	14.04	14.03	14.05	14.04	14.03	14.07	14.05	14.05
SID Lys	0.85	0.85	0.85	0.73	0.73	0.73	0.61	0.61	0.61
SID Met	0.24	0.24	0.24	0.22	0.22	0.22	0.22	0.22	0.22
SID Thr	0.52	0.52	0.52	0.46	0.46	0.46	0.43	0.43	0.43
SID Trp	0.15	0.15	0.15	0.13	0.13	0.13	0.13	0.13	0.13
ME/CP	0.99	0.99	0.99	0.99	0.99	0.98	0.99	0.98	0.98
SID Lys/ME	0.06	0.06	0.06	0.05	0.05	0.05	0.04	0.04	0.04
SID Lys/SID Met	3.55	3.48	3.52	3.32	3.36	3.29	2.75	2.81	2.72
SID Lys/SID Thr	1.63	1.62	1.63	1.58	1.59	1.58	1.40	1.42	1.43
SID Lys/SID Trp	5.55	5.57	5.55	5.48	5.45	5.48	4.54	4.57	4.55

^a^*CP* Crude protein, *FVS Flammulina velutipes* stem waste, *Lys* Lysine, *ME* Metabolizable energy, *Met* Methionine, *SID* Standardized ileal digestible, *Thr* Threonine, *Trp* Tryptophan

^b^Vitamin mineral premix provided the following quantities per kg of diet: vitamin A, 6000 IU; vitamin D_3_, 2400 IU; vitamin E, 20 IU; vitamin K_3_, 2 mg; vitamin B_1,_ 0.96 mg, vitamin B_2_, 4 mg, vitamin B_6_, 2 mg, vitamin B_12_, 12 μg; pantothenic acid, 11.2 mg; niacin, 22 mg; choline chloride, 80 mg; folacin, 0.4 mg; biotin, 40 μg; Mn, 12 mg (MnO); Fe, 76 mg (FeSO_4_·H_2_O); Zn, 76 mg (ZnO); Cu, 120 mg (CuSO_4_·5H_2_O); I, 0.24 mg (KI); Se, 0.40 mg (Na_2_SeO_3_)

^c^These values were calculated from data provided in Exp. 1 and 2

All pigs were housed in pens with drinkers, feeders and slatted floors, and were given free access to water and feed. The environment temperature of the barn was controlled between 25 and 29 °C, and relative humidity was controlled at 60–70%. The experiment lasted for 9 weeks, including 3 phases: growing phase (d 1 to 17), growing-finishing phase (d 18 to 37) and finishing phase (d 38 to 63). Pigs and feed were weighed at the beginning of the experiment (d 1), and the end of each phase (d 17, 37 and 63) to determine average daily gain (ADG), average daily feed intake (ADFI) and gain to feed ratio (G/F).

The blood and fecal samples were acquired as described by Zhao et al. [11]. From d 16 to 17, d 36 to 37 and d 62 to 63, approximately 100 g of fresh feces was collected from each pen for 2 d, and then the fecal samples were immediately stored at − 20 °C. All the samples were pooled by pen and then dried at 65 °C for 72 h. Fresh feces were collected directly from a rectum on d 17, d 37 and d 63, and immediately stored in liquid nitrogen until analysis for SCFAs.

Blood samples were collected by venepuncture in the morning (08:00) of d 17, d 37 and d 63 after overnight fasting, and then were injected into a 10-mL vacuum tube. After centrifugation at 3000 × *g* for 15 min (4 °C), the serum samples were harvested and stored at − 20 °C for further biochemical analyses. All samples were collected from pigs with the closest body weight (BW) to average BW of each pen.

At the end of the trial, 12 pigs close to the average BW from each pen were selected (2 gilts and 2 barrows per treatment). After 12 h fasting, the pigs were electronically stunned (250 V, 0.5 A, for 5-6 s), exsanguinated and eviscerated by the standard commercial procedure.

After the pigs were killed, the gastrointestinal tract of each pig was ligated, jejunal mucosa were scraped by a glass slide and then stored in liquid nitrogen until western blot analyses were done. Then, duodenal, jejunal and ileal samples of approximately 3 cm in length were stored in paraformaldehyde solution (4%) for a microscopic assessment of the mucosal morphology. Finally, colonic and caecal digesta were collected and immediately frozen in liquid nitrogen for later microbial population determination.

About 10 g of *longissimus dorsi* muscle (LDM) on the left half of each carcass were sampled and then stored at − 20 °C for measurements of intramuscular fat concentration, amino acid profile and fatty acids profile.

### Chemical analysis

All the diets, feces and digesta samples collected in animal trials were ground to pass through a 1-mm screen and mixed thoroughly before chemical analysis. All the diets, feces and digesta samples were analyzed for crude protein (CP, procedure 984.13; AOAC) [12], dry matter (DM, procedure 930.15; AOAC) [12], ether extract (EE, procedure 920.39; AOAC) [12], ash (procedure 942.05; AOAC) [12], neutral detergent fiber (NDF) and acid detergent fiber (ADF) [13]. The concentrations of NDF and ADF were analyzed using fiber bags and fiber analyzer equipment (200, Ankom Technology Corp., Macedon, NY, USA). In addition, all the diets, feces, and urine samples collected were analyzed for gross energy (GE) using an Oxygen Bomb Calorimeter (6300, Parr Instruments, Moline, USA). In Exp. 2, the 18 AA concentrations in diets, feces and digesta samples were determined following the standard methods in AOAC. Specifically, with the exception of methionine, cysteine and tryptophan, the AA concentrations were determined after hydrolysis with 6 mol/L HCl at 110 °C for 24 h using an AA analyzer (Hitachi L-8900, Tokyo, Japan). Methionine and cysteine were determined as methionine sulphone and cysteic acid using an AA analyzer (Hitachi L-8900, Tokyo, Japan) after cold performic acid oxidation overnight and hydrolyzing with 7.5 mol/L HCl at 110 °C for 24 h. Tryptophan was determined using High Performance Liquid Chromatography (Agilent 1200 Series, Santa Clara, CA, USA) after LiOH hydrolysis for 22 h at 110 °C. The chromium concentration in diets used in Exp. 2 and ileal digesta were determined using a polarized Zeeman Atomic Absorption Spectrometer (Hitachi Z2000, Tokyo, Japan) after nitric acid-perchloric acid wet ash sample preparation according to the procedure of Williams et al. [14]. In Exp. 3, fresh fecal samples were thawed at 4 °C and processed according to the procedure of Wu et al. [15], and then the concentrations of SCFA were determined via ion chromatography (ICS 3000, Thermo Crop., CA, USA).

### Biochemical index assay in serum

The concentrations of serum immunoglobulin A (IgA), immunoglobulin G (IgG) and immunoglobulin (IgM) were determined using the ELISA test kits (immunoglobulins quantitation kit; Nanjing Jiancheng Bioengineering Institute, Nanjing, China). The levels of antioxidant parameters including superoxide dismutase (SOD), glutathione peroxidase (GSH-Px), total antioxidant capacity (T-AOC) and malondialdehyde (MDA) were determined using assay kits following the manufacturer’s instructions (Nanjing Jiancheng Bioengineering Institute, Nanjing, China). The interleukin-2 (IL-2) concentration was determined using commercially available porcine ELISA kits (Nanjing Jiancheng Bioengineering Institute, Nanjing, China). The levels of low-density lipoprotein (LDL), high-density lipoprotein (HDL), growth hormone (GH), triglyceride (TG) and total cholesterol (TC) in serum were determined using automatic biochemical analyzer (7170, Hitachi Corp., Tokyo, Japan) with corresponding kits (Nanjing Jiancheng Bioengineering Institute, Nanjing, China).

### Carcass characteristics determination

After slaughter, hot carcass weight was recorded immediately, and the dressing percentage of the individual pig was calculated through dividing the carcass weight by the live body weight. Carcass straight length was measured from the first rib to the end of the public bone. Carcass oblique length was measured between the first rib and sternum and the pubic symphysis midline [16]. Backfat thickness and loin-eye area at the 10^th^ rib was measured according to the Chinese Guidelines on Performance Measurement Technology and Regulations for Pigs [17]. Briefly, backfat thickness was measured using a vernier caliper, and three points were recorded: the first rib, last rib and last lumbar vertebra. Loin eye height and width were measured, and then the loin eye area was calculated following the equation: loin eye area (cm^2^) = 0.7 × loin eye height (cm) × loin eye width (cm).

### Meat quality assessment

The LDM on the left half of each carcass between the 10^th^ and 12^th^ ribs was sampled for further assessment on meat quality, including the following parameters: the muscle color, pH, shear force, drip loss, cooking loss and marbling score. Meet color, including ⊿L* (lightness), ⊿a* (redness) and ⊿b* (yellowness) was measured three times at 24 h postmortem using a tristimulus colorimeter (CR-410, Konica minota, Tokyo, Japan). At 45 min postmortem, an incision was made on the LDM and initial muscle pH_45min_ was measured with a glass penetration pH electrode (pH-star, DK 2730, Herlev, Denmark), and the pH_24h_ was detected 24 h postmortem in the chilling room (4 °C). The shear force of LDM was measured according to the procedures described by Ciobanu et al. [18]. Specifically, each sample was previously cooked in a water bath at 70 °C for 20 min, and then ten cylindrical samples (10 mm diameter × 10 mm length) were obtained from cutting the meat parallel to the fiber orientation, and determined by cutting the sample vertically to the myofiber axis using a digital-display-muscle tenderness meter (C-LM3B, Tenovo, Harbin, China). Drip loss was measured as described previously by Straadt et al. [19]. Briefly, approximately 30 g of meat slice was hung in a plastic bag at 4 °C for 24 h, and the meat was kept out of contact with the bag. Drip loss was calculated as a percentage of the amount of drip compared to the initial weight. Lastly, marbling score of LDM was determined according to the NPPC Guidelines [20], in which a 1 to 10-point scale (1 = devoid, 10 = abundant) was applied to evaluate the subjective marbling score.

Cooking procedures were according to Aaslyng et al. [21]: Each steak was weighed alone just before placing them in bags, then put them in oven at 80 °C. When the temperature reached 80 °C in the center of each steak, the roast ras removed from the oven, and the liquid in the bag was removed. The processed roast was weighed, then the cooking loss was calculated.

### Intramuscular fat, amino acids and fatty acids profile of LDM

About 20 g of each meat sample was cut into thin slices (2 to 3 mm), weighted in an aluminum box, and then put into a vacuum frozen dryer (Tofflon Freezing Drying Systems, Shanghai, China) to freeze-dry for 72 h. Lyophilized meat was subsequently crushed into powder, and the intramuscular fat concentration was measured by Soxhlet petroleum ether extraction (XT15 Extractor, Ankom Technology Corp., Macedon, NY, USA) as described by Zhang et al. [22]. Moreover, the AA concentrations in LDM samples were determined using the same procedure as determining the AA concentrations in samples of Exp. 2. The fatty acids profile was determined using classical gas chromatography (6890 series, Agilent Technologies, Wilmington, DE, USA) as described by Sukhija et al. [23].

### Western blot assay

Western blot assay was conducted to determine the relative expression of occludin, zonula occludens-1 (ZO-1) and claudin-1 in ileal samples of the individual pigs (*n* = 3) following the procedures described by Kansagra et al. [24] and Zhang et al. [25]. Briefly, frozen ileal mucosal tissues were homogenized in 2 mL protein lysis buffer, and the protein concentration of the supernatant fractions was quantified by a bicinchoninic acid protein assay kit (02912E, Cowin Biotech Co., Ltd., Beijing, China). Then equal protein amounts were separated on SDS-PAGE gels, and separated proteins were transferred onto nitrocellulose membranes. After blocking in tris-buffered saline (containing 3% non-fat dry milk), blots were incubated overnight at 4 °C with specific primary antibodies, including rabbit polyclonal anti zonula occludens-1 (NBP1–85047, Novus Biologicals, CO, USA), rabbit polyclonal anti occludin (Ab216327, Abcam company, UK) and rabbit polyclonal anti claudin-1 (NBP1–77036, Novus Biologicals, CO, USA). After three times washes with tris-buffered saline (containing 0.1% Tween 20), blots were reacted for 1 h with a horseradish peroxidase-conjugated secondary antibody (goat anti rabbit IgG, HRP 111–035-003, Jackson ImmunoResearch Laboratories, Inc. PA, USA), washed, and incubated in detection reagent, and the images were captured. Blots were also probed with anti-actin antibodies.

### Duodenal, jejunal and ileal morphology

The duodenal, jejunal and ileal samples were douched with physiologic saline and then stored in paraformaldehyde solution (4%). The preserved segments were prepared after staining with hematoxylin-eosin (HE) solution according to the standard paraffin-embedding procedures. Morphometric variables, including villus height and crypt depth were measured with an image processing and analysis system (Image Pro Plus 7.0, Media Cybernetics Inc., Bethesda, MD, USA), and then the V/C was calculated.

### Bacterial DNA extraction, PCR amplification, and Illumina MiSeq sequencing

Microbial community genomic DNA was extracted from colon and cecum digesta using the Fast DNA® Spin Kit (MP Biomedicals, Irvine, CA, USA) following the manufacturer’s recommendations. The bacterial DNA extract was checked using 1% agarose gel, and DNA concentration and purity were determined with UV-vis spectrophotometer (NanoDrop 2000, Thermo Scientific, Wilmington, USA). The V3 hypervariable region of 16S rRNA gene were amplified with primer pairs 338F (5′ – ACTCCTACGGGAGGCAGCAG – 3′), 806R (5′ – GGACTACHVGGGTWTCTAAT – 3′) by a PCR themocycler (GeneAmp 9700, ABI, CA, USA). The PCR amplification conditions included: initial denaturation at 95 °C for 3 min, followed by 27 cycles of denaturing at 95 °C for 30 s, and then annealing at 55 °C for 30 s, extension at 72 °C for 45 s, single extension at 72 °C for 10 min, and ended at 4 °C. The PCR product was excised from 2% agarose gel and purified by AxyPrep DNA Gel Extraction Kit (Axygen Biosciences, Union City, CA, USA). Purified amplicons were pooled in equimolar and paired-end sequenced on an Illumina MiSeq platform (Illumina Company, San Diego, USA) following the standard protocols. Sequences with an average quality score lower than 20, the truncated reads shorter than 50 bp, and containing ambiguous bases were discarded. Only overlapping sequences longer than 10 bp were assembled according to their overlap. Subsequently, data of the sequences was conducted for the cluster analysis of operational taxonomic units via Usearch (Version 8.1.1861, http://www.drive5.com/usearch) and Qiime (Version 1.8, http://qiime.org) [26].

### Calculation

In Exp. 1, the DE and ME values in FVS was calculated based on the difference method and following the equation described by Adeola [27]: DE (MJ/kg, as-fed basis) = (GE_I_ – GE_F_) / DM_I_; ME (MJ/kg, as-fed basis) = (GE_I_ – GE_F_ – GE_U_) / DM_I_, where GE_I_, GE_F_, GE_U_ and DM_I_ are gross energy intake, output in feces, output in urine, and matter intake respectively. The ATTD of GE, CP, DM, OM, EE, NDF and ADF in FVS were calculated according to the equation obtained from Kong et al. [5]: D_ti_ (%) = [D_td_ - D_bd_ × (1 - P_ti_)] / P_ti_, where D_ti_, D_td_ and D_bd_ are the concentration of the nutrients in the test ingredient, test diet and basal diet, respectively, and P_ti_ is the proportion of the test ingredient in the experimental diet.

In Exp. 2, the AID of AAs of FVS was calculated using the following the equation described by Stein et al. [28]: AID (%) = 100 - [(AA_digesta_ / AA_diet_) × (Cr_diet_ / Cr_digesta_) × 100], where AA_digesta_ and Cr_digesta_ are the concentrations of AA and chromium in the digesta, respectively, and AA_diet_ and Cr_diet_ are the concentrations of AA and chromium in the experiemental diet, respectively. The basal ileal endogenous loss of each AA (IAA_end_, g/kg of DM intake) for each pig fed the N free diet was calculated following the equation: IAA_end_ = [AA_digesta_ × (Cr_diet_ / Cr_digesta_)]. The SID of AAs of FVS was calculated using the following equation: SID (%) = [AID + (IAA_end_ / AA_diet_) × 100].

In Exp. 3, the ATTD of nutrients were calculated according to the equation described by Kong et al. [5]: Digestibility (%) = 100 – [(CI_in_ × CC_out_ / CI_out_ × CC_in_) × 100], in which CI_in_ and CI_out_ are the concentrations of chromic oxide in diets and feces, respectively, and CC_in_ and CC_out_ are the nutrient contents in diets and feces, respectively.

### Statistical analysis

Data of growth performance, nutrient digestibility, SCFA concentrations, serum biochemical indices, carcass traits, meat quality, intestinal morphological indexes and microflora data were checked for normality and outliers using the PROC UNIVERIATE procedure of SAS 9.2 (SAS Inst. Inc., Carry, NC, USA). Outliers were identified using cook’s distance and abandoned when analyzing data. Then data were analyzed using the PROC GLM procedure of SAS. The treatment diet was the only fixed effect and each pig was treated as the experimental unit (for growth performance data, each pen was treated as the experimental unit). The LSMEANS statement was used to separate treatment means, with Tukey’s test for adjustment. Significant differences were declared at *P* < 0.05.

## Results

### Energy concentration and AA digestibility of FVS

The DE and ME values of FVS were 4.11 and 3.64 MJ/kg **(**as-fed basis), respectively. The ATTD of DM, OM, GE and CP in FVS were 73.3%, 77.2%, 71.5%, and 61.4%, respectively, while the ATTD of NDF and ADF in FVS were 36.4% and 32.2%, respectively (Table 4). The AID and SID values of the indispensable AAs and CP in FVS ranged from 14.9% to 59.0% and 17.50% to 59.47%, respectively (Table 5).

**Table 4:** Apparent total tract digestibility of nutrients and available energy concentration (MJ/kg) in *Flammulina velutipes* stem waste (Exp. 1)^a^

Item	FVS
Apparent total tract digestibility, %	
Dry matter	73.3
Gross energy	71.5
Crude protein	61.4
Organic matter	77.2
Neutral detergent fiber	36.4
Acid detergent fiber	32.2
Available energy, MJ/kg, as–fed basis	
Gross energy	15.88
Digestible energy	4.11
Metabolizable energy	3.64
Available energy, MJ/kg, dry matter basis	
Gross energy	17.70
Digestible energy	4.58
Metabolizable energy	4.06

^a^Values are the means of 6 observations. *FVS Flammulina velutipes* stem waste

**Table 5:** Apparent ileal digestibility and standardized ileal digestibility of crude protein and animo acids in *Flammulina velutipes* stem waste (%, dry-matter basis, Exp. 2) ^a^

Item	AID value	SID value
Crude protein	25.4	48.7
Indispensable amino acids		
Lysine	19.0	21.3
Methionine	38.7	41.0
Threonine	24.5	28.5
Tryptophan	37.9	44.1
Valine	30.9	34.4
Leucine	44.8	46.0
Isoleucine	14.9	17.5
Phenylalanine	59.0	59.5
Histidine	26.7	29.9
Arginine	47.9	50.8
Dispensable amino acids		
Tyrosine	56.6	56.8
Serine	26.0	29.5
Glutamic acid	59.1	60.5
Proline	−201.6	−195.5
Glycine	−28.5	−19.7
Alanine	53.9	56.4
Cysteine	−13.4	−7.2
Aspartic acid	35.6	38.1

^a^*AID* Apparent ileal digestibility, *SID* Standardized ileal digestibility. AID values of crude protein and amino acids were calculated using the exogenous indicator method, and values for SID were calculated by correcting the AID values with the basal endogenous losses. In the current study, the averaged basal endogenous losses for crude protein, lysine, methionine, threonine, tryptophan, valine, lecuine, isoleucine, phenylalanine, histidine, arginine, tyrosine, serine, glutamic acid, proline, glycine, alanine, cycstine and aspartic acid measured through animal trials were 14.00, 0.50, 0.12, 0.81, 0.37, 0.60, 0.63, 0.36, 0.36, 0.26, 0.54, 0.16, 0.66, 0.90, 1.16, 1.50, 0.60, 0.31 and 0.90 g/kg dry matter intake, respectively, all within the reseanable ranges reported previsously [29]

### Growth performance and apparent total tract digestibility (ATTD) of nutrients of pigs fed FVS diets

Pigs at 50 to 75 kg fed a diet with 5% FVS revealed markedly decreased ADG (*P* < 0.01), ADFI (*P* < 0.01) and ATTD of OM (*P* = 0.04) compared with the control diet and 2.5% FVS diet (Tables 6 and 7). ATTD of GE (*P* < 0.05) were higher in pigs fed a diet with 2.5% FVS than those fed the 5% FVS diet. Pigs fed diet with FVS enhanced ATTD of EE (*P* < 0.01), NDF (*P* < 0.01) and ADF (*P* < 0.01) compared with pigs fed the control diet. However, there was no difference in G/F (*P* = 0.99) during this period.

**Table 6:** Effects of dietary *Flammulina velutipes* stem waste inclusion on growth performance of growing-finishing pigs (Exp. 3)

Item	Control	2.5% FVS	5% FVS	SEM	*P*-value
BW d 0, kg	63.9	64.0	64.0	0.4	0.970
Body weight 50 to 75 kg					
ADG, g	987.5^a^	930.2^a^	834.9^b^	27.3	0.009
ADFI, g	2479.6^a^	2347.9^a^	2113.5^b^	51.7	0.002
G/F	0.40	0.40	0.40	0.01	0.987
Body weight 75 to 100 kg					
ADG, g	954.0	873.2	895.3	23.7	0.089
ADFI, g	2990.7^a^	2792.8^b^	2724.4^b^	53.5	0.014
G/F	0.32	0.31	0.33	0.01	0.190
Body weight 100 to 135 kg					
ADG, g	823.5	885.4	864.6	33.5	0.322
ADFI, g	2914.4	2918.7	2827.7	85.0	0.134
G/F	0.28	0.30	0.31	0.01	0.386
Overall growing-finishing phase					
ADG, g	909.2	893.6	866.4	17.9	0.259
ADFI, g	2821.3^a^	2724.7^ab^	2602.2^b^	55.1	0.025
G/F	0.32^b^	0.33^sb^	0.34^a^	0.01	0.024

^a-c^Least squares means within different superscripts differ (*P*<0.05)

*ADFI* Average daily feed intake, *ADG* Average daily gain, *BW* Body weight, *FVS Flammulina velutipes* stem waste, *G/F* Gain to feed ratio, *SEM* Standard error of the mean

**Table 7:** Effects of dietary *Flammulina velutipes* stem waste inclusion on apparent total tract digestibility of nutrients of growing-finishing pigs (%, Exp. 3)

Item	Control	2.5% FVS	5% FVS	SEM	*P*-value
Body weight 50 to 75 kg					
Dry matter	83.6	84.0	82.2	0.5	0.067
Organic matter	87.1^a^	87.3^a^	85.7^b^	0.4	0.035
Gross energy	83.6^ab^	84.2^a^	82.2^b^	0.5	0.048
Crude protein	74.4	75.2	72.2	0.8	0.066
Ether extract	16.0^b^	50.8^a^	49.6^a^	2.8	<0.001
Neutral detergent fiber	45.2^b^	56.6^a^	62.9^a^	2.3	0.001
Acid detergent fiber	36.8^b^	55.4^a^	61.9^a^	2.4	<0.001
Body weight 75 to 100 kg					
Dry matter	85.2^a^	84.9^a^	82.2^b^	0.6	0.010
Organic matter	88.3^a^	88.1^a^	85.8^b^	0.5	0.015
Gross energy	85.1^a^	85.2^a^	82.3^b^	0.6	0.014
Crude protein	78.4^a^	77.7^a^	68.7^b^	1.4	0.001
Ether extract	34.4^c^	61.4^a^	49.4^b^	1.6	<0.001
Neutral detergent fiber	55.9	52.9	55.1	2.7	0.728
Acid detergent fiber	53.0	55.8	57.7	3.2	0.594
Body weight 100 to 135 kg					
Dry matter	86.6^a^	85.1^b^	85.1^b^	0.3	0.003
Organic matter	89.5^a^	88.2^b^	88.4^b^	0.2	0.003
Gross energy	86.3^a^	84.9^b^	85.0^b^	0.3	0.009
Crude protein	78.1	78.9	78.3	0.7	0.709
Ether extract	88.1^a^	51.1^b^	51.1^b^	1.2	<0.001
Neutral detergent fiber	57.1^c^	61.9^b^	66.8^a^	1.2	<0.001
Acid detergent fiber	59.3	61.9	63.0	1.1	0.111

^a-c^Least squares means within different superscripts differ (*P*<0.05). *FVS Flammulina velutipes* stem waste, *SEM* Standard error of the mean

Dietary FVS inclusion decreased the ADFI (*P* = 0.01) in pigs at 75 to 100 kg. Apparent total tract digestibility of DM (*P* = 0.01), OM (*P* = 0.02), GE (*P* = 0.01) and CP (*P* < 0.01) were less with 5% dietary FVS inclusion compared with control and 2.5% FVS diet. The ATTD of EE in pigs fed diet with 2.5% FVS was higher (*P* < 0.01) compared with the control diet and 5% FVS diet during this phase. In addition, no significant changes on ADG and G/F was noted during this period.

For pigs at 100 to 135 kg, there were no effect of dietary FVS inclusion on ADG (*P* = 0.32), ADFI (*P* = 0.13), G/F (*P* = 0.39), and ATTD of CP (*P* = 0.71) and ADF (*P* = 0.11). However, decreased ATTD of DM (*P* < 0.01), OM (*P* < 0.01), GE (*P* = 0.01) and EE (*P* < 0.01) and increased ATTD of NDF (*P* < 0.01) was observed in pigs fed the FVS diets. During the overall growing-finishing phase, pigs consumed the 5% FVS diet showed markedly decreased ADFI (*P* = 0.03), increased G/F (*P* = 0.02) and unchanged ADG (*P* = 0.26) compared to pigs fed the control diet.

### Short chain fatty acid (SCFA) concentration in fresh feces

The concentrations of acetate (*P* < 0.01), formate (*P* < 0.01), butyrate (*P* = 0.01), isovalerate (*P* < 0.01) and total SCFA (*P* < 0.01) in feces of pigs consumed the FVS diet were elevated on d 17 compared with those fed the control diet (Table 8). The propionate (*P* = 0.01) concentration of pigs fed the 5% FVS diet and valerate (*P* = 0.02) concentration of pigs fed the 2.5% FVS diet were higher than the other two treatments on d 17.

**Table 8:** Effects of dietary *Flammulina velutipes* stem waste inclusion on short chain fatty acid concentration measured in fresh feces of pigs in different growing-finishing phases (mg/kg) (Exp. 2)

Item	Control	2.5% FVS	5% FVS	SEM	*P*-value
d 17					
Lactate	22.4	69.8	110.1	25.6	0.099
Acetate	3941.9^b^	4790.8^a^	5353.8^a^	179.2	< 0.001
Propionate	3069.8^b^	3219.2^b^	4021.2^a^	188.3	0.011
Formate	73.4^b^	161.9^a^	165.3^a^	16.8	0.005
Isobutyrate	2348.9	2285.4	2533.2	306.1	0.841
Butyrate	67.6^b^	278.2^a^	351.3^a^	49.2	0.006
Isovalerate	175.3^b^	387.6^a^	392.2^a^	35.1	0.002
Valerate	355.8^b^	721.8^a^	305.4^b^	90.6	0.017
Total	10,055.0^b^	11,914.6^a^	13,232.4^a^	476.4	0.003
d 37					
Lactate	284.0	276.2	53.8	65.7	0.054
Acetate	4432.7^b^	4417.6^b^	5384.5^a^	170.7	0.004
Propionate	2948.1^b^	3084.7^b^	4124.3^a^	135.4	< 0.001
Formate	71.1^b^	159.1^a^	142.3^a^	12.6	0.001
Isobutyrate	1624.5^b^	2360.5^ab^	2651.2^a^	242.5	0.035
Butyrate	109.6^c^	476.6^a^	294.8^b^	44.6	0.001
Isovalerate	194.5^b^	562.1^a^	462.2^a^	48.9	0.001
Valerate	454.0	487.7	468.4	77.9	0.954
Total	10,118.5^c^	11,824.7^b^	13,581.3^a^	461.6	0.001
d 63					
Lactate	166.4^b^	787.8^a^	895.5^a^	181.4	0.036
Acetate	4421.7	5221.1	4650.6	283.9	0.173
Propionate	2946.5	3464.7	3368.5	217.5	0.248
Formate	65.5^b^	211.1^a^	233.1^a^	40.6	0.031
Isobutyrate	1840.1	2057.0	2393.7	158.0	0.089
Butyrate	105.2^c^	315.7^b^	560.6^a^	53.6	0.001
Isovalerate	281.8	424.0	510.8	61.8	0.071
Valerate	465.5	288.5	223.4	65.3	0.063
Total	10,292.5^b^	12,769.9^a^	12,836.3^a^	580.5	0.017

^a-c^Least squares means within different superscripts differ (*P*<0.05)

*FVS Flammulina velutipes* stem waste, *SEM* Standard error of the mean

On d 37, pigs fed the 5% FVS diet showed increased concentrations of acetate (*P* < 0.01) and propionate (*P* < 0.01) compared with the control and the 2.5% FVS diet, increased concentration of isobutyrate (*P* = 0.04) compared with the control diet, and decreased concentration of butyrate (*P* < 0.01) compared with the 2.5% FVS diet. Moreover, compared with the control diet, the concentration of formate (*P* < 0.01), butyrate (*P* < 0.01), isovalerate (*P* < 0.01) and total SCFA (*P* < 0.01) on d 37 and the concentration of lactate (*P* = 0.04), formate (*P* = 0.03), butyrate (*P* < 0.01) and total SCFA (*P* = 0.02) on d 63 all markedly increased with the inclusion of dietary FVS.

### Serum biochemical profiles

As shown in Table 9, no significant changes on concentrations of IgA, IgG, IgM, SOD, MDA, IL-2, HDL, LDL, TC, TG and GH in serum of pigs fed the FVS diet were observed relative to the control diet in all phases. However, dietary FVS inclusion improved the GSH-Px activity in d 17 (*P* < 0.01) and d 37 (*P* = 0.01), and also increased (*P* = 0.03) the T-AOC level in d 63.

**Table 9:** Effects of dietary *Flammulina velutipes* stem waste inclusion on serum profile of pigs in different growing-finishing phases (Exp. 3)

Item	Control	2.5% FVS	5% FVS	SEM	*P*-value
d 17					
IgA, g/L	17.48	20.51	25.32	2.91	0.220
IgG, g/L	9.23	11.01	14.77	1.98	0.191
IgM, g/L	9.76	10.67	12.82	1.91	0.535
GSH-Px, U/mL	125.75^b^	168.73^a^	173.27^a^	6.87	0.002
SOD, U/mL	48.64	45.06	47.20	1.13	0.142
T-AOC, U/mL	13.19	14.62	14.00	0.77	0.458
MDA, nmol/mL	1.10	1.22	1.28	0.12	0.588
IL-2, pg/mL	30.64	33.12	29.91	2.17	0.572
HDL, mmol/L	1.10	1.01	0.99	0.05	0.274
LDL, mmol/L	1.05	1.09	1.13	0.07	0.720
TC, mmol/L	2.40	2.41	2.45	0.11	0.939
TG, mmol/L	0.56	0.69	0.74	0.07	0.216
GH, ng/mL	2.10	2.03	2.21	0.15	0.689
d 37					
IgA, g/L	18.49	17.56	16.87	1.95	0.844
IgG, g/L	9.83	9.88	8.54	0.88	0.505
IgM, g/L	10.52	10.02	9.37	1.08	0.759
GSH-Px, U/mL	165.65^b^	192.32^a^	183.65^a^	4.72	0.012
SOD, U/mL	49.38	49.40	48.08	0.59	0.253
T-AOC, U/mL	14.88	13.42	12.78	0.64	0.120
MDA, nmol/mL	1.11	1.10	1.10	0.02	0.859
IL-2, pg/mL	41.09	55.01	46.41	6.87	0.396
HDL, mmol/L	0.91	0.89	0.97	0.04	0.363
LDL, mmol/L	1.02	0.94	1.08	0.09	0.534
TC, mmol/L	2.22	2.12	2.41	0.10	0.178
TG, mmol/L	0.66	0.65	0.79	0.04	0.066
GH, ng/mL	1.96	1.71	1.82	0.15	0.515
d 63					
IgA, g/L	24.01	27.5	25.55	2.55	0.641
IgG, g/L	13.54	15.31	16.98	1.81	0.443
IgM, g/L	13.06	14.88	15.6	1.65	0.561
GSH-Px, U/mL	151.29	172.65	143.91	10.49	0.193
SOD, U/mL	47.98	49.03	47.83	0.92	0.622
T-AOC, U/mL	11.00^b^	13.21^a^	13.25^a^	0.53	0.027
MDA, nmol/mL	1.13	1.14	1.11	0.02	0.585
IL-2, pg/mL	32.46	33.16	38.70	3.50	0.425
HDL, mmol/L	1.05	0.87	1.03	0.07	0.161
LDL, mmol/L	1.10	1.15	1.20	0.07	0.655
TC, mmol/L	2.49	2.31	2.60	0.11	0.226
TG, mmol/L	0.74	0.62	0.82	0.06	0.118
GH, ng/mL	2.37	2.70	2.30	0.25	0.512

^a-b^Least squares means within a row with different superscripts differ (*P*<0.05)

*FVS Flammulina velutipes* stem waste, *GH* Growth hormone, *GSH-Px* Glutathione peroxidase, *HDL* High density lipoprotein, *IgA* Immunoglobulin A, *IgG* Immunoglobulin G, *IgM* Immunoglobulin M, *IL-2* Interleukin-2, *LDL* Low density lipoprotein, *MDA* Malondialdehyde, *SEM* Standard error of the mean, *SOD* Superoxide dismutase, *T-AOC* Total antioxidant capacity, *TC* Total cholesterol, *TG* Triglyceride

### Carcass characteristics and meat quality

Pigs fed the 5% FVS diet revealed decrease in backfat thickness (*P* = 0.03; Table 10) and tended to decrease the dressing percentage (*P* = 0.06) compared with those fed the control diet. The other carcass traits including the hot carcass weight, carcass length and loin-eye area, and the meat quality including meat color, pH, shear force, drip loss, cooking loss, marbling score and intramuscular fat were not influenced by dietary FVS inclusion.

**Table 10:** Effects of dietary *Flammulina velutipes* stem waste inclusion on carcass characteristics and meat quality of finishing pigs (Exp. 3)

Item	Control	2.5% FVS	5% FVS	SEM	*P*-value
Carcass traits					
Hot carcass weight, kg	95.38	92.45	89.43	1.55	0.081
Dressing percentage, %	73.02	71.60	71.86	0.38	0.060
Carcass straight length, cm	105.00	102.50	103.88	0.91	0.205
Carcass oblique length, cm	92.88	90.38	90.63	1.04	0.229
Backfat thickness, mm	15.75^a^	16.50^ab^	12.75^b^	0.86	0.030
Loin-eye area, cm^2^	35.18	36.97	35.77	3.67	0.946
Meat quality					
L* (lightness)	55.86	55.02	56.88	1.16	0.550
a* (redness)	19.58	18.75	18.42	0.52	0.318
b* (yellowness)	7.10	7.12	7.46	0.44	0.814
pH_45min_	6.12	6.22	6.28	0.04	0.126
pH_24h_	5.74	5.81	5.67	0.05	0.237
Shear force, kg	32.25	23.55	27.42	2.53	0.143
Drip loss, %	3.81	3.19	5.36	0.88	0.252
Cooking loss, %	25.43	24.27	24.67	0.99	0.714
Marbing score (1–10 scale)	1.75	1.50	1.25	0.24	0.367
Intramuscular fat, %	2.92	2.81	2.40	0.41	0.656

^a-b^Least squares means within a row with different superscripts differ (*P*<0.05)

Values are means with pooled SEM, *n* = 4. *FVS Flammulina velutipes* stem waste, *pH*_*45min*_ pH at 45 min after postmortem, *pH*_*24h*_ pH at 24 h after postmortem, *SEM* Standard error of the mean

### Amino acids and fatty acids profiles in the LDM

Diets supplemented with FVS did not change the AA concentrations in LDM, but 5% dietary FVS addition increased the concentrations of alpha-linolenic acid (*P* = 0.02) and ∑ n-3 PUFA (*P* = 0.02) as well as the PUFA / SFA ratio (*P* = 0.03) in LDM compared with control diet and 2.5% FVS diet (Tables 11 and 12). In addition, the ratio of n-6/n-3 was markedly decreased (*P* = 0.02) in LDM of pigs fed the FVS diets.

**Table 11:** Effects of dietary *Flammulina velutipes* stem waste inclusion on amino acids profile measured in the *longissimus dorsi* muscle of finishing pigs (%, Exp. 3)

Item	Control	2.5% FVS	5% FVS	SEM	*P*-value
Indispensable amino acids					
Lysine	7.67	7.66	7.75	0.13	0.879
Methionine	2.57	2.62	2.72	0.05	0.229
Threonine	3.93	3.95	3.99	0.07	0.796
Tryptophan	0.93	0.94	0.96	0.02	0.614
Valine	4.17	4.20	4.20	0.08	0.964
Leucine	6.83	6.84	6.89	0.12	0.918
Isoleucine	3.98	3.98	4.04	0.07	0.819
Phenylalanine	3.35	3.36	3.39	0.06	0.889
Histidine	3.98	3.91	4.00	0.12	0.857
Arginine	5.35	5.30	5.36	0.09	0.917
Dispensable amino acids					
Tyrosine	2.98	2.98	3.03	0.05	0.782
Serine	3.39	3.40	3.44	0.06	0.860
Glutamic acid	12.29	12.18	12.44	0.15	0.545
Proline	3.12	3.15	3.14	0.05	0.908
Glycine	3.57	3.58	3.56	0.07	0.976
Alanine	4.76	4.75	4.79	0.08	0.942
Cysteine	0.88	0.92	0.93	0.02	0.392
Aspartic acid	7.91	7.87	7.97	0.12	0.815
Total amino acids	81.66	81.59	82.60	1.38	0.855

Values are means with pooled SEM, *n* = 4. *FVS Flammulina velutipes* stem waste, *SEM* Standard error of the mean

**Table 12:** Effects of dietary *Flammulina velutipes* stem waste inclusion on fatty acids profile in the *longissimus dorsi* muscle of finishing pigs (mg/g, of fresh tissue) (Exp. 3)

Item	Control	2.5%FVS	5%FVS	SEM	*P*-value
Saturated fatty acid					
Caproic acid (C6: 0)	0.16	0.14	0.15	0.01	0.246
Capric acid (C10: 0)	0.16	0.16	0.15	0.02	0.861
Lauric acid (C12: 0)	0.09	0.09	0.07	0.01	0.747
Myristic acid (C14: 0)	1.34	1.24	1.09	0.23	0.751
Pentadecanoic acid (C15: 0)	0.02	0.03	0.03	0.01	0.063
Palmitic acid (C16: 0)	23.89	21.44	20.00	3.97	0.788
Heptadecanoic acid (C17: 0)	0.35	0.13	0.16	0.14	0.529
Stearic acid (C18: 0)	12.41	12.28	10.72	2.34	0.853
Icosanoic acid (C20: 0)	0.24	0.21	0.19	0.05	0.777
Heneicosanoic acid (C21: 0)	0.33	0.29	0.37	0.04	0.345
Docosanoic acid (C22:0)	0.06	0.08	0.08	0.01	0.561
Tetracosanoic acid acid (C24: 0)	0.03	0.04	0.04	0.01	0.689
Monounsaturated fatty acid					
Myristoleic acid (C14: 1)	0.03	0.02	0.02	0.01	0.582
Palmitoleic acid (C16: 1)	3.38	2.68	2.42	0.45	0.364
Oleic acid (C18: 1n-9c)	39.84	32.66	33.36	6.17	0.680
Eicosenoic acid (C20: 1)	0.76	0.55	0.55	0.15	0.525
Nervonic acid (C24: 1)	0.08	0.05	0.06	0.02	0.684
Polyunsaturated fatty acid					
Linoleic acid (C18: 2n-6c)	7.16	7.39	9.64	0.81	0.132
Alpha-linolenic acid (C18: 3n-3)	0.22^b^	0.29^b^	0.45^a^	0.04	0.025
Dihomo-γ-linolenic (C20: 3n-6)	0.22	0.18	0.21	0.01	0.230
Arachidonic acid (C20: 4n-6)	1.49	1.32	1.59	0.10	0.273
Eicosatrienoic acid (C20: 3n-3)	0.05	0.05	0.08	0.01	0.156
Eicosapentanoic acid (C20: 5n-3)	0.05	0.06	0.05	0.02	0.843
Erucic acid (C22: 1n-9)	0.03	0.03	0.02	0.01	0.657
Docosadienoic acid (C22: 2)	0.05	0.04	0.03	0.01	0.107
Docosahexaenoic acid (C22: 6n-3)	0.10	0.09	0.06	0.02	0.314
∑ SFA	39.09	36.13	33.02	6.63	0.816
∑ MUFA	44.09	35.96	36.42	6.76	0.654
∑ PUFA	9.36	9.45	12.17	0.88	0.108
∑ n-6 PUFA	8.87	8.89	11.44	0.83	0.114
∑ n-3 PUFA	0.41^b^	0.49^b^	0.67^a^	0.05	0.021
n-6/n-3	21.93^a^	18.4^b^	17.29^b^	0.81	0.016
PUFA/SFA	0.26^b^	0.26^b^	0.38^a^	0.03	0.025

^a-b^ Least squares means within a row with different superscripts differ (*P*<0.05)

Values are means with pooled SEM, *n* = 4. *FVS Flammulina velutipes* stem waste, *MUFA* Monounsaturated fatty acid, *PUFA* Polyunsaturated fatty acid, *SEM* Standard error of the mean, *SFA* Saturated fatty acid

### Western blot assay

**Fig. 1:**
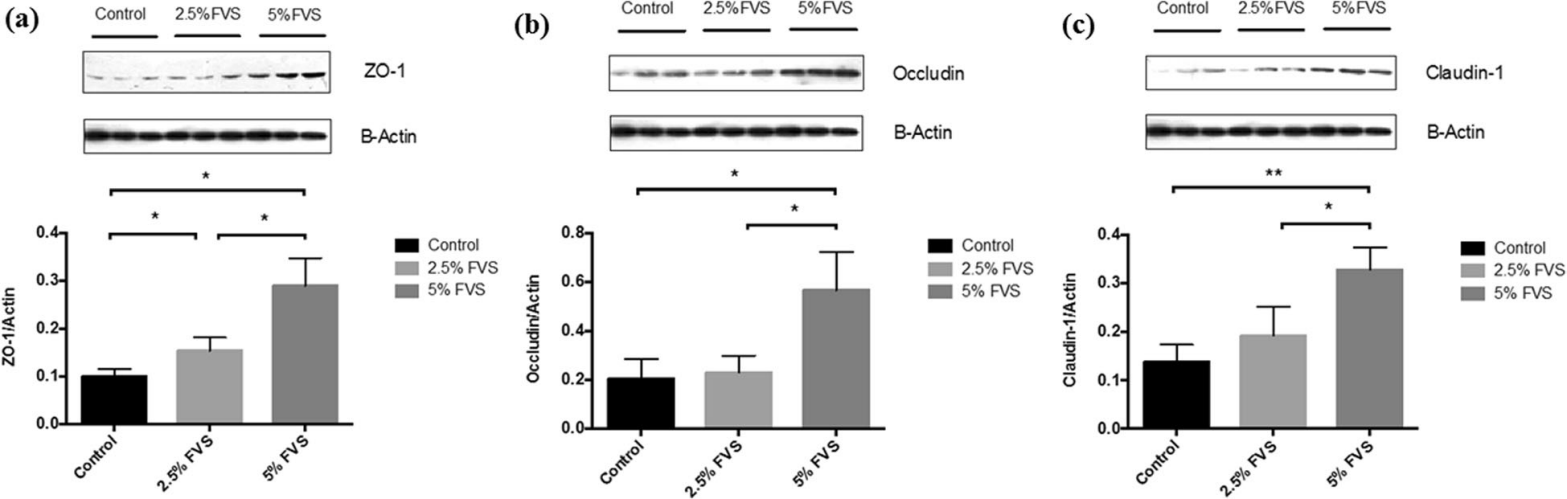
Effects of dietary *Flammulina velutipes* stem waste (FVS) inclusion on the relative expression of tight junction proteins in jejunum of growing pigs. **a** The relative expression of ZO-1 in jejunum of growing pigs fed control and 2.5% or 5% FVS diets. **b** The relative expression of occludin in jejunum of growing pigs fed control and 2.5% or 5% FVS diets. **c** The relative expression of claudin-1 in jejunum of growing pigs fed control and 2.5% or 5% FVS diets. Values are means (3 pigs per treatment) with standard errors represented by vertical bars. *P < 0.05, **P < 0.01

Dietary FVS inclusion increased the protein expression of occluding (*P* < 0.01), ZO-1 (*P* < 0.01) and claudin-1 (*P* < 0.01) in the ileal mucosal tissues compared with the control diet (Fig. 1).

### Intestinal morphological indexes

**Fig. 2:**
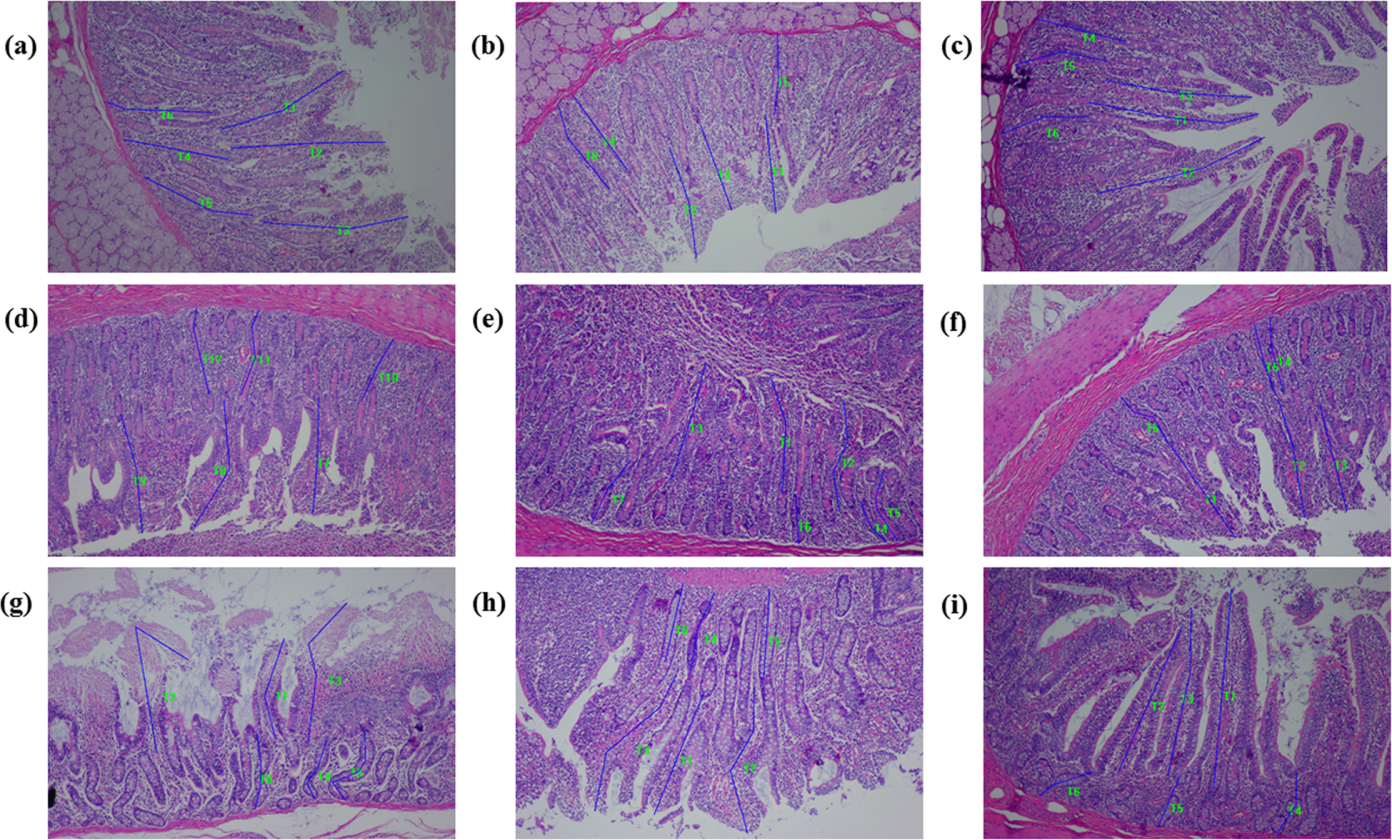
Intestinal morphology of growing pigs fed the control diet or diets with 2.5% or 5% *Flammulina velutipes* stem waste (FVS) inclusion. **a** Representative duodenum morphology of growing pigs fed the control diet. **b** Representative duodenum morphology of growing pigs fed diet supplied with 2.5% FVS. **c** Representative duodenum morphology of growing pigs fed diet supplied with 5% FVS. **d** Representative jejunum morphology of growing pigs fed the control diet. **e** Representative jejunum morphology of growing pigs fed diet supplied with 2.5% FVS. **f** Representative jejunum morphology of growing pigs fed diet supplied with 5% FVS. **g** Representative ileum morphology of growing pigs fed the control diet. **h** Representative ileum morphology of growing pigs fed diet supplied with 2.5% FVS. **i** Representative ileum morphology of growing pigs fed diet supplied with 5% FVS. T1-T12 represents the villus height or crypt depth measured

Diets supplemented with 2.5% FVS increased (*P* < 0.01) the ratio of V/C in jejunum in comparison with the control and 5% FVS diets (Table 13 and Fig. 2).

**Table 13:** Effects of dietary *Flammulina velutipes* stem waste inclusion on the intestinal mucosa morphology of finishing pigs (Exp. 3)

Item	Control	2.5% FVS	5% FVS	SEM	*P*-value
Duodenum					
Villus height, μm	278.18	326.97	290.79	12.60	0.077
Crypt depth, μm	208.49	206.96	220.19	17.33	0.084
V/C	1.41	1.61	1.35	0.12	0.343
Jejunum					
Villus height, μm	213.40	243.15	217.15	12.59	0.267
Crypt depth, μm	171.65	151.61	165.38	10.74	0.451
V/C	1.27^b^	1.64^a^	1.37^b^	0.03	0.001
Ileum					
Villus height, μm	268.23	285.91	326.18	14.34	0.070
Crypt depth, μm	189.61	160.61	191.44	22.11	0.573
V/C	1.50	1.88	1.87	0.13	0.136

^a-b^Least squares means within a row with different superscripts differ (*P*<0.05)

Values are means with pooled SEM, *n* = 4. *FVS Flammulina velutipes* stem waste, *SEM* Standard error of the mean, *V/C* the ratio of villus height to crypt depth

### Intestinal microbiome composition

**Fig. 3:**
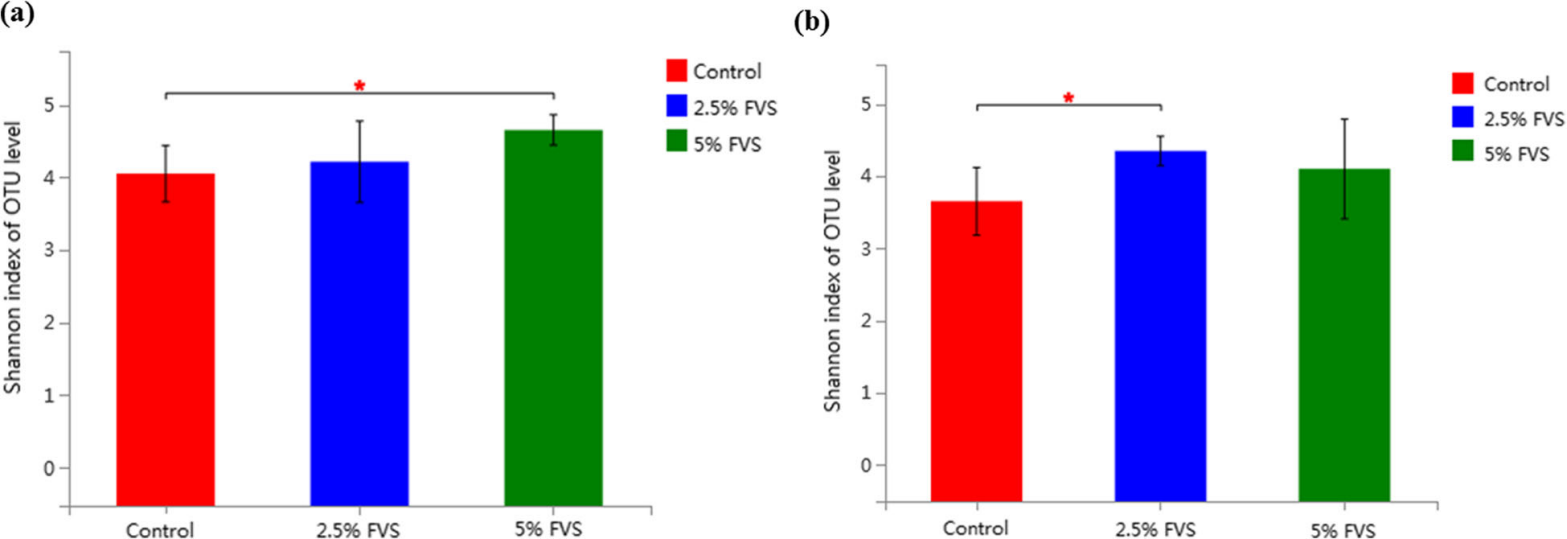
Alpha-diversity of microbiota measured by Shannon index observed in the caecal digesta (**a**) and colonic digesta (**b**) of finishing pigs fed diets with 0, 2.5% and 5% *Flammulina velutipes* stem waste (FVS). (*n* = 4 for each treatment). Error bars are shown as SEM and *P*-values are from Student’s t-test. *P < 0.05

**Fig. 4:**
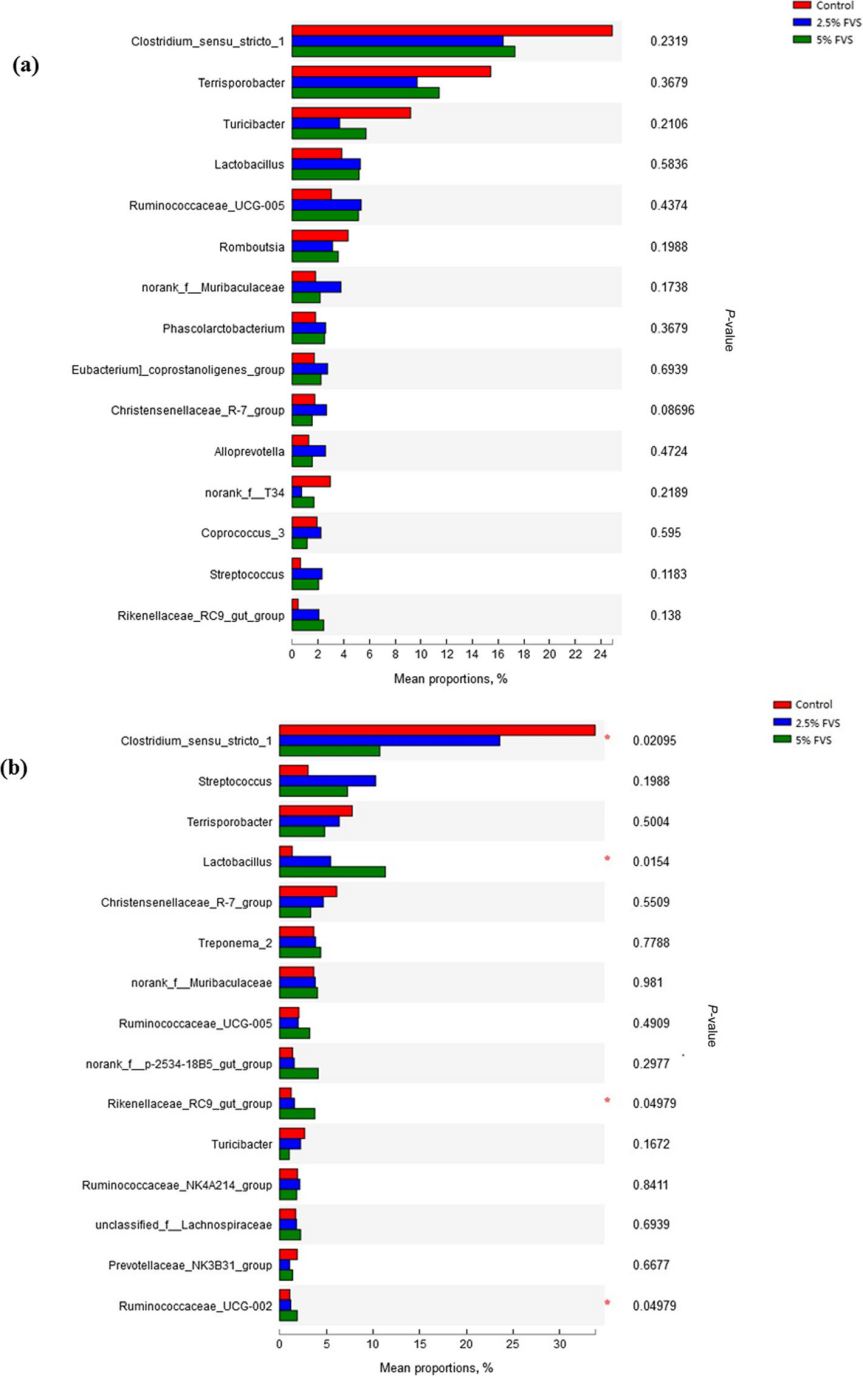
Relative abundances of genera among samples from caecal digesta (**a**) and colonic digesta (**b**) of finishing pigs fed diets with 0, 2.5 and 5% *Flammulina velutipes* stem waste (FVS). A Kruskal-Wallis H test was used to evaluate the significance of differences between the control diet and diets with 2.5% or 5% FVS inclusion. *P < 0.05

Diets with 2.5% and 5% FVS addition strikingly improved the microbiota diversity compared with the control diet in caecum (*P* = 0.03) and colon (*P* = 0.01), respectively (Fig. 3). There was no difference on microbial populations between different diets in caecum of finishing pigs. However, in the colon, a decrease in the *Clostridium-sensu-stricto-1* populations (*P* = 0.02), and an increase in the *Lactobacillus* (*P* = 0.02)*, Rikenellaceae-RC9-gut-group* (*P* < 0.05) and *Ruminococcaceae-UCG-002* (*P* < 0.05) populations (Fig. 4) were found in pigs fed FVS diet compared with those fed the control diet.

## Discussion

### Available energy values and amino acid digestibility

The NDF and ADF concentrations of FVS were greater than those of corn bran (NDF: 32.96%; ADF: 9.23%) and wheat bran (NDF: 32.28%; ADF: 11.00%), but much less than those of soybean hulls (NDF: 59.39%; ADF: 41.55%), indicating that FVS is a fibrous ingredient. The DE and ME values in FVS were less than some other fibrous ingredients, such as wheat bran (DE: 10.13 MJ/kg, ME: 9.70 MJ/kg), and soybean hulls (DE: 8.40 MJ/kg, ME: 8.11 MJ/kg) [10]. Moreover, the digestibility of CP and AAs of FVS were also much less than other fibrous ingredients, such as wheat bran (AID of CP: 69%, AID of AAs: range from 51% to 84%, SID of CP: 78%, SID of AAs: range from 56% to 90%), and soybean hulls (AID of CP: 44%, AID of AAs: range from 34% to 74%, SID of AAs: range from 54% to 82%) [10].

The low energy and nutrients digestibility of FVS may partly due to its high dietary fiber concentration, which had a negative effect on energy and nutrients digestibility [30]. There were negative SID and AID values of cysteine (Cys), proline (Pro) and glycine (Gly) in pigs fed the FVS diet, which indicated a higher endogenous flow of those AAs at the distal ileum compared to the absorbed contents from the diet. Because FVS was the only AA source in the experimental diet in Exp. 2, the tissue of pigs had to release AAs to synthesize protein when the protein required for maintainence cannot be obtained from FVS [31]. Moreover, the high levels of Gly and Pro in ileal digesta of pigs fed FVS may also be related to the secretion of bile and saliva, considering the large amounts of Gly and Pro in saliva and bile, which was consistent with the previous studies [32]. In addition, FVS contains high NDF and ADF concentrations, and the dietary fiber inclusion also led to higher endogenous losses of Gly and Pro in pigs [33].

### Effects of dietary FVS inclusion on growth performance and nutrient digestibility of pigs

Liu et al. [2] demonstrated that 2.5% FVS inclusion in diets had no negative effects on growth performance but impared ATTD of nutrients in piglets, which were similar to the reults in growing-finishing pigs in the current study except for the ADFI results. Song et al. [34] reported that dietary inclusion with fermented oyster mushroom by-products decreased ADG in finishing pigs. Our study also showed that pigs at 50 to 75 kg fed higher level of FVS (5%) significantly impaired the ADG which was consistent with previous study, and there was no effect for diet with 2.5% FVS inclusion compared with control diet.

According to Oriol et al. [35], dietary fiber inclusion was an important factor that could affect the palatability and feed intake of pigs. Obviously, except for pigs at 100 to 135 kg, diets supplied with FVS affected the ADFI of pigs in the current study, which is consistent with most previous studies that have shown the negative effects of high levels of dietary fiber inclusion on voluntary feed intake and growth performance in growing pigs [36, 37]. There was no effect for ADFI of pigs at 50 to 75 kg fed diet with 2.5% FVS compared with control diet, but a significantly decreased ADFI was observed when pigs fed diet with 5% FVS, which indicated that higher level of FVS (5%) negatively affected the palatability during this period. In addition to the fiber components, a more likely explanation of the negative effects of FVS on pig growth performance is the palatability of FVS. Gromwell et al. [38] showed that pigs fed diets with 45% corn distillers dried grains with solubles (DDGS, with more than 20% NDF) had minimal effects on growth performance of pigs. In the current study, FVS was included up to 5% and the dietary NDF levels only ranged from 10.99% to 13.55%, much less than the NDF level used in the previous corn DDGS study. As a result, the parlatability of FVS may be the major challenge for the wide utilization of this ingredient in swine diets.

Wenk [39] and Berrocoso et al. [40] indicated that high fiber diets had a negative effect on digestibility of nutrients and energy. In this study, with the dietary FVS inclusion, the ATTD of OM and GE significantly decreased during every phase, while the ATTD of DM and CP in pigs at 75 to 100 kg and the ATTD of DM and EE in in pigs at 100 to 135 kg also significantly decreased. However, the nutrient digestibility in pigs fed the 2.5% FVS diet were not different from the control diet, which indicated that less inclusion of FVS (2.5%) did not affect these nutrient digestibility during those periods. These results were partly agreed with some previous studies, e.g. Wilfart et al. [37] found that increased dietary fiber level significantly decreased the ATTD of DM, OM, CP and GE, but the ATTD of dietary fiber was unaffected. In addition, Bindelle et al. [41] reported that when the inclusion level of dietary fiber increased from 9.6 to 25.4%, the ATTD of DM, OM and CP of growing pigs linearly decreased, but the ATTD of NDF linearly increased. The different basal diets and fiber sources may lead to different reports. Högberg [42] also pointed out that the effects of dietary fiber levels on nutrient digestibility may differ with the properties of the fiber. As Choi et al. [43] reported, NDF rather than the other dietary fiber types as an independent variable could increase the accuracy of digestible energy prediction in feeds for growing pigs.

### Effects of dietary FVS inclusion on SCFA concentration and serum profile of pigs

The SCFA concentrations in feces of pigs fed the FVS diet significantly increased during the growing-finishing phase. Previous studies indicated that the carbohydrate profiles of mushrooms include different types of low-digestible and non-digestible carbohydrates, such as β-glucans, chitin, oligosaccharides and resistant starch, suggesting that they may stimulate the SCFA concentration and impact gut microbial populations [44]. Chitin concentrations in *Flammulina velutipes* were 7.7%, and was greater than that in the other mushrooms [45]. The carbohydrate such as chitin in *Flammulina velutipes* may play a key role in increasing the SCFA concentrations in hindgut of pigs.

Glutathione peroxidase is a group of selenoenzymes in which selenium is an integral part [46]. The dietary selenium intake is essential for the antioxidant enzyme defences, and FV has been found to contain 2.8 μg/kg selenium [47], which may play an important role in elevating the GSH-Px concentrations. In addition, increased T-AOC was observed in pigs fed the FVS in the current study, which further suggested the antioxidant-promoting effect of dietary FVS. Therefore, FVS may be a potential candidate to enhance the antioxidant capacity of growing-finishing pigs according to our results.

### Effects of dietary FVS inclusion on carcass characteristics, meat quality and LDM chemical composition of finishing pigs

Stahly et al. [48] showed that dietary fiber addition resulted in depressed dressing percentage, and we observed that ingestion of the fibrous FVS tended to decrease the dressing percentage compared with pigs fed the normal diet, which was also in accordance with the reports by Chu et al. [49], who indicated that the dressing percentage significantly decreased in pigs consumed the fermented mushroom by-product diets. It was highly likely that greater gut fill of finishing pigs fed high fiber diets that led to the reduced dressing percentage [50].

Li et al. [51] showed that dietary ramie, an ingredient with high fiber concentration, decreased backfat depth in finishing pigs, which is consistent with our study. Our study provided the evidence that dietary FVS inclusion markedly reduced the backfat depth of pigs, which demonstrated the role of FVS in decreasing subcutaneous fat accumulation and improving carcass characteristics. Previous study has found that finishing pigs fed high fiber caused less backfat [52], which may also explain part of our results.

The fatty acid profiles of pork have been a primary area of consumer concern. Adequate intake of n-3 PUFA, a balanced n-6/n-3 ratio, even a proper PUFA / SFA ratio, may reduce the risk of life-style diseases such as inflammatory and immune disorders [53], hypertension [54], coronaryartery disease [55] and diabetes [56].

Alpha-linolenic acid is an essential omega-3 fatty acid, and is very important for a wide range of functions in the immune system, cardiovascular system and endocrine system of humans. Enser et al. [57] indicated that the alpha-linolenic acid (18:3n-3) are more easily influenced by diets compared with the saturated and monounsaturated fatty acids in pigs, because the alpha-linolenic acid cannot be synthesized *in vivo*. Therefore, the significantly improved alpha-linolenic acid concentration in the current study may reflect the dietary change caused by 5% dietary FVS inclusion.

The main components of adipose tissue are fatty acids, and the essential fatty acids including n-6 and n-3 polyunsaturated fatty acid (PUFA) cannot be converted into each other in animal’s body. Therefore, PUFA are crucial components of the food [58]. Both n-6 and n-3 PUFA can regulate the relative gene expression: n-6 PUFA can increase the concentrations of inflammatory mediators, while n-3 PUFA exert suppressive effects on chronic diseases [59, 60]. Previous studies suggested that diets with high ratios of n-6/n-3 ratio may increase the inflammatory mediators’ production and lead to the metabolic syndrome, such as Alzheimer’s disease, cognitive impairment and type 2 diabetes [61–63]. Therefore, a higher n-6/n-3 ratio in diets is harmful for the health of humans. In this study, 5% dietary FVS inclusion not only elevated the value of n-3 PUFA, but also decreased the ratio of n-6/n-3. Less dietary FVS addition level (2.5%) could only reduce the n-6/n-3 ratio. Thus, moderate dietary FVS inclusion could partly change the PUFA compositions of the pork, which is beneficial for the health of humans. In addition, the ratio of PUFA / SFA obviously increased with 5% dietary FVS inclusion in the current study, which means that the SFA concentration declined while the PUFA concentration improved with the increased level of dietary FVS. Meat with a less n-6/n-3 PUFA ratio and rich in n-3 PUFA is also beneficial for human consumption. Therefore, we suggested that dietary FVS inclusion in pigs may be a good practice to improve the nutritional quality of lipids in pork products.

### Effects of dietary FVS inclusion on intestinal tight junction proteins expression, morphology and microflora of finishing pigs

Lallès [64] disclosed that the gastrointestinal tract plays an important role as a physiological barrier between the body and the outer environment, and in process of food digestion and nutrient absorption. Intestinal permeability had been considered as an important indicator of the intestinal epithelial barrier function, and this barrier function was primarily regulated by a system of an epithelial junctional complex, which is referred to as the tight junction [65, 66]. The paracellular barrier function of the intestinal epithelia is considered to be regulated by several types of tight junction (TJ) proteins, such as occludin, ZO-1 and claudin-1 [67, 68]. In Fanning’ point of view [69], occludin, ZO-1 and claudin-1, the main structural barrier proteins, are the most critical and important components in the functional and structural organization of the tight junctions. As shown in our study, FVS-treated pigs revealed a significant increase in the expression of those three TJ proteins. Considering the fact that TJ proteins play a critical role in intestinal epithelial barrier integrity [70], we suggested that dietary FVS inclusion could improve intestinal integrity in pigs, which was evident from the increased expression of occludin, ZO-1 and claudin-1.

Montagne et al. [71] suggested that the villus height to crypt depth ratio represents the absorption capacity of the small intestine. In this study, the ratio of V/C in the jejunum of pigs in 2.5% FVS group was increased relative to those in the control and 5% FVS groups, which suggested that 2.5% FVS inclusion in diets could influence the intestinal absorption capacity of pigs by regulating the morphological structures of the intestine.

Mosca et al. [72] revealed that many human diseases are associated with the loss of microbial diversity in the gut microbiota. Our results showed that finishing pigs consumed diets with 2.5% and 5% FVS inclusion increased the microbiota diversity compared with control diet in caecum and colon, respectively. Dietary FVS addition could partly improve the healthy status of finishing pigs by enhancing microbial diversity in hindgut.

Our study indicated that *Clostridium_sensu_stricto_1* was the predominant genus in the colon of pigs, but its proportion depressed with the dietary FVS inclusion. Previous study [73] showed that the proportion of *Clostridium_sensu_stricto_1* sharply reduced when the dietary crude protein level decreased, which may be due to the shortage of protein substrate for fermentation. In that case, in this study, the reduction of the proportion of *Clostridium_sensu_stricto_1* may due to the less digestible CP contents by dietary FVS inclusion.

*Lactobacillus* is one of the major bacterial groups in porcine gastrointestinal tract, and is a most commonly used probiotic agent. *Lactobacillus* could benefit intestinal health by stimulating the growth of the healthy microbiota, and preventing the colonization of enteric pathogens in intestine, thus improving the digestive ability of pigs [74]. We observed that the *Lactobacillus* proportion was significantly increased by the dietary FVS inclusion, and may suggest that dietary FVS could promote the finishing pigs’ gut health to some extend by elevate the abundance of *Lactobacillus.*

As described by Huang et al. [75], Rikenellaceae and *Ruminococcus* are the predominantly SCFA-producing genus, which could ferment carbohydrates and produce acetate and propionate. Dietary FVS inclusion enhanced the proliferation of the relative SCFA-producing bacteria, which is in accordance with our results that FVS could increase the SCFA concentration in fresh feces.

In general, these results demonstrated that moderate FVS inclusion can partly promote the development of the small intestine, microbiota diversity, and optimize the beneficial intestinal microbiota in growing-finishing pigs.

## Conclusions

Although FVS-treated pigs showed impaired growth performance to some extent, dietary FVS inclusion could improve gut health, antioxidant capacity in serum, carcass trait and meat quality. Overall, despite its low available energy and AA digestibility, FVS could be served as a promising alternative fibrous ingredient in diets fed to growing-finishing pigs.

## Data Availability

All data generated or analyzed during this study are included in this published article.

## Abbreviations

AAs: Amino acids
ADFI: Average daily feed intake
ADF: Acid detergent fiber
ADG: Average daily gain
AID: Apparent ileal digestibility
ATTD: Apparent total tract digestibility
CP: Crude protein
DE: Digestible energy
DM: Dry matter
EE: Ether extract
FV: Flammulina velutipes
FVS: *Flammulina velutipes* stem waste
GE: Gross energy
G/F: Feed conversion efficiency
GH: Growth hormone
GSH-Px: Glutathione peroxidase
HDL: High-density lipoprotein
IBW: Initial body weight
IgA: Immunoglobulin A
IgG: Immunoglobulin G
IgM: Immunoglobulin M
IL-2: Interleukin-2
IL-4: Interleukin-4
LDL: Low-density lipoprotein
LDM: longissimus dorsi muscle
MDA: Malondialdehyde
ME: Metabolizable energy
NDF: Neutral detergent fiber
OM: ORGANIC matter
PUFA: Polyunsaturated fatty acid
SCFA: Short chain fatty acid
SFA: Saturated fatty acid
SID: Standardized ileal digestibility
SOD: Superoxide dismutase
T-AOC: Total antioxidant capacity
TC: Total cholesterol
TG: Triglyceride
TJ: Tight junction
V/C: Villus height to crypt depth ratio
ZO-1: Zonula occludens-1
